# Augmented binary method for basket trials (ABBA)

**DOI:** 10.1177/09622802251403365

**Published:** 2025-12-05

**Authors:** Svetlana Cherlin, James M S Wason

**Affiliations:** Population Health Sciences Institute, Newcastle University, Newcastle upon Tyne, UK

**Keywords:** augmented binary method, basket clinical trials, Bayesian inference, immune-mediated inflammatory diseases, latent variable framework

## Abstract

In several clinical areas, traditional clinical trials often use a responder outcome, a composite endpoint that involves dichotomising a continuous measure. An augmented binary method that improves power while retaining the original responder endpoint has previously been proposed. The method leverages information from the undichotomised component to improve power. We extend this method for basket trials, which are gaining popularity in many clinical areas. For clinical areas where response outcomes are used, we propose the new augmented binary method for basket trials that enhances efficiency by borrowing information on the treatment effect between subtrials. The method is developed within a latent variable framework using a Bayesian hierarchical modelling approach. We investigate the properties of the proposed methodology by analysing point estimates and high-density intervals in various simulation scenarios, comparing them to the standard analysis for basket trials that assumes binary outcomes. Our method results in a reduction of 95% high-density interval of the posterior distribution of the log odds ratio and an increase in power when the treatment effect is consistent across subtrials. We illustrate our approach using real data from two clinical trials in rheumatology.

## 1. Introduction

In many clinical trials for complex diseases, selecting a single primary endpoint has proven challenging, with many endpoints being of clinical interest.^1^ Responder endpoints are commonly proposed to combine distinct components into a single measure. These are typically composite endpoints consisting of binary and at least one continuous measure. The continuous components are often dichotomised using a pre-specified threshold. Patients are classified as responders if their continuous component reaches the threshold, in addition to meeting the criteria for the binary component(s). For example, in the context of cancer trials, a patient is defined as a responder if their tumour size shrinks by more than 30% and they do not have new tumour lesions detected.^2^ However, it has been shown that using binary responder endpoints leads to larger expected and maximum sample sizes compared to continuous endpoints.^3^ To this end, the augmented binary method^4^ has been developed to model the joint distribution of the continuous outcome and a binary outcome. The method improves the precision of analysis and increases power by avoiding the loss of information from dichotomising a continuous component. Initially developed for cancer trials, the method has since been adapted for use in clinical trials for rheumatoid arthritis.^5^

In the area of rheumatic diseases, the most common outcomes are the disease activity score, which includes different 28-joint counts (DAS28),^6^and the American College of Rheumatology (ACR) improvement criterion^7^; both are based on dichotomised information such as DAS28 being less than a specific threshold, or ACR achieving a pre-specified percentage threshold. Additionally, a binary indicator b of whether or not the patients received rescue medications or discontinued treatment is often used, known as ‘non-responder imputation’.

Patients are considered responders based on both binary or dichotomised continuous variables, and the binary indicator b . The augmented binary method does not dichotomise the outcome measure. Instead, it analyses the continuous outcome (DAS28 or ACR), and the binary indicator b described above. By jointly modelling continuous and binary outcomes, the method utilises information on the distance from the dichotomisation threshold for the continuous outcome. The augmented binary method outperformed the standard method that analyses binary endpoints in rheumatoid arthritis, as is evident by a substantial reduction in the 95% confidence intervals for the probability of success without inflation in type I error rate, indicating that the estimated probability of response is more precisely estimated. The augmented binary method has been extended to incorporate multiple follow-up times^8^ and multiple doses of the same treatment within the latent variable framework.^9,10^ Additionally, the original augmented binary method has been implemented in a Bayesian framework.^11^

However, the method has so far been restricted to standard single-arm and two-arm clinical trials. Recently, basket trials, which aim to investigate a single therapy across multiple conditions, have become popular.^12^ Basket trials consist of subtrials characterised by a specific disease, with each subtrial investigating the same treatment. Originating in the field of oncology and aiming to target shared molecular aberrations, basket trials have been extended to other areas such as immune-mediated inflammatory diseases (IMIDs), where similar symptoms are shared across multiple conditions.^13^ The efficiency of basket trials lies in their requirement of fewer patients and a shorter duration compared to traditional trial designs, owing to the borrowing of information between subtrials. It has been demonstrated that sharing information between the subtrials in a basket trial leads to more efficient utilisation of data.^14–18^

In this work, we extend the sharing of information to composite outcomes in basket trials, while leveraging the augmented binary method via a latent variable framework. This method is motivated by basket trials, which are most commonly used in oncology and typically analyse RECIST response.^19^ We propose that responder outcomes in basket trials in IMIDs could be modelled similarly to trials in oncology. The method is based on a latent variable approach and is implemented in a Bayesian framework. It utilises a multivariable normal distribution to model the continuous outcome and a latent variable that is assumed to determine the binary indicator b . By avoiding dichotomisation of the outcome, we aim to achieve more precise inference and increased power of the trial. We illustrate the method using clinical trials in rheumatology where the same responder endpoint is used. Since it is challenging to obtain individual data for basket trials in an academic setting, we illustrate the method’s application using a hypothetical basket trial constructed from two distinct trials for which we were able to obtain data. Although the example is not an actual basket trial but rather two separate trials that could potentially have been run as a basket trial, the application of the method is by no means narrow. The method could be useful in the majority of basket trials, as they are most commonly used in oncology with RECIST response as a primary outcome. In addition, there are other clinical areas where basket trials are promising (but as of yet immature in application) and responder outcomes are used, such as immune-mediated inflammatory diseases.

## 2. Methods

### 2.1. Notation

Suppose a randomised, placebo-controlled trial is conducted, in which patients i=1,…,N are equally randomised to treatment ( $t_{i}=1$ ) or control ( $t_{i}=0$ ). At the end of the trial, two outcomes are measured for each patient: a continuous score, such as a DAS28 or an American College of Rheumatology N (ACR-N) score ( $y_{i1}$ ) and a binary indicator for receiving rescue medication ( $y_{i2}$ ).

We assume that we are working with a 20% improvement in the American College of Rheumatology criteria (ACR20) response criteria, where a response is defined if the ACR-N is dichotomised at 20%. We work on the log scale, as this was shown to be closer to normally distributed in previous work.^5^ According to this criterion, a patient is defined as a responder if

$$y_{i1}\geq \log(20)\;\mathrm{and}\;y_{i2}=0$$

We assume that there is a continuous latent variable $y_{i2}^{⋆}$ that determines whether a patient receives rescue medication or is a non-responder for another pre-defined reason:

$$y_{i2}=1(y_{i2}^{⋆}>0)$$

where 1 is an indicator function taking the value 1 if its argument is true and 0 otherwise. Thus, the patient is a responder if

$$y_{i1}>\log(20)\;\mathrm{and}\;y_{i2}^{⋆}>0$$

### 2.2. Stratified augmented binary method

We introduce an augmented binary method for a non-basket framework based on a latent variable approach (hereafter referred to as *stratified analysis*). The outcomes are modelled using a multivariate probit regression model as follows:

(1) $$\begin{array}{rl} y_{i1} & =\mu_{i1}+\epsilon_{i1},\;y_{i2}^{⋆}=\mu_{i2}+\epsilon_{i2}\\ (\epsilon_{i1},\epsilon_{i2}{)}^{T} & ∼\text{MVN}\{0,\Sigma );\;\Sigma =\left(\begin{array}{cc} \sigma_{1}^{2} & \sigma_{1}\sigma_{2}\rho \\ \sigma_{2}\sigma_{1}\rho & \sigma_{2}^{2} \end{array}\right) \end{array}$$

We decompose the covariance matrix Σ as Σ=diag(σ)×Ω×diag(σ) , where Ω is the correlation matrix

$$\Omega =\left(\begin{array}{cc} 1 & \rho \\ \rho & 1 \end{array}\right)$$

and σ is the vector of standard deviations $\sigma =(\sigma_{1},\sigma_{2})$ . For details on implementing the multivariate probit regression model approach, see Section 2.6. The standard deviation of the latent variable, $\sigma_{2}$ , is set to 1 for identifiability reasons.

The means of the outcomes are modelled as follows:

$$\begin{array}{rl} \mu_{i1} & =\beta_{1}+\gamma_{1}\log(x_{i})+\theta_{1}t_{i}\\ \mu_{i2} & =\beta_{2}+\gamma_{2}\log(x_{i})+\theta_{2}t_{i} \end{array}$$

where $x_{i}$ is the baseline measure for some disease activity score or any other clinically important covariate for patient i , $\beta =(\beta_{1},\beta_{2})$ , $\gamma =(\gamma_{1},\gamma_{2})$ and $\theta =(\theta_{1},\theta_{2})$ are vectors of the intercepts, the effects of the baseline measure and the treatment effect, respectively.

Based on previous research on borrowing information in basket trials,^17^the parameters for the intercept and baseline effect, $\beta =(\beta_{1},\beta_{2})$ , $\gamma =(\gamma_{1},\gamma_{2})$ are assigned independent normal priors $N(0,5^{2})$ . The treatment effects, $\theta =(\theta_{1},\theta_{2})$ , are assigned independent normal priors $N(0,10^{2})$ . Thus, prior 95% high density intervals (−9.8,9.8) and (−19.6,19.6) cover a wide range of possible β , γ , and θ . The individual level standard deviation of the continuous outcome is given an inverse gamma prior $\sigma_{1}∼\text{IG}(0.5,0.005)$ . The correlation matrix is given a Lewandowski, Kurowicka, and Joe (LKJ) prior^20^ Ω∼LKJCorr(η) with a shape parameter η=5 . The shape parameter was chosen to allow for a wide range of correlations.

### 2.3. Augmented binary method for basket trials (ABBA)

We now extend the method to cover a randomised, placebo-controlled basket trial with K subtrials. We assume a total of N patients are included in the study with K subtrials ( i=1,…,N) . Within each subtrial k=1,…,K , the patients are equally randomised to treatment ( $t_{i}=1$ ) or control ( $t_{i}=0$ ), and the outcomes are collected and modelled similarly to the stratified augmented binary model (see equation (1)).

The means of the outcomes are modelled as follows:

$$\begin{array}{rl} \mu_{i1} & =\beta_{k1}+\gamma_{k1}\log(x_{i})+\theta_{k1}t_{i}\\ \mu_{i2} & =\beta_{k2}+\gamma_{k2}\log(x_{i})+\theta_{k2}t_{i} \end{array}$$

where $\beta_{k}=(\beta_{k1},\beta_{k2})$ , $\gamma_{k}=(\gamma_{k1},\gamma_{k2})$ and $\theta_{k}=(\theta_{k1},\theta_{k2})$ are vectors representing the intercepts, the effects of the baseline measure, and the treatment effect for subtrial k , respectively.

#### 2.3.1. Prior level 1

The vectors $\beta_{k}=(\beta_{k1},\beta_{k2})$ , $\gamma_{k}=(\gamma_{k1},\gamma_{k2})$ and $\theta_{k}=(\theta_{k1},\theta_{k2})$ for subtrial k have independent multivariate normal priors:

$$\begin{array}{rl} \beta_{k} & ∼\text{MVN}(\mu_{\beta },\Sigma_{\beta })\\ \gamma_{k} & ∼\text{MVN}(\mu_{\gamma },\Sigma_{\gamma })\\ \theta_{k} & ∼\text{MVN}(\mu_{\theta },\Sigma_{\theta }) \end{array}$$

where

$$\begin{array}{rl} \Sigma_{\beta } & =\left(\begin{array}{cc} \sigma_{\beta 1}^{2} & 0\\ 0 & \sigma_{\beta 2}^{2} \end{array}\right),\\ \Sigma_{\gamma } & =\left(\begin{array}{cc} \sigma_{\gamma 1}^{2} & 0\\ 0 & \sigma_{\gamma 2}^{2} \end{array}\right)\\ \Sigma_{\theta } & =\left(\begin{array}{cc} \sigma_{\theta 1}^{2} & 0\\ 0 & \sigma_{\theta 2}^{2} \end{array}\right) \end{array}$$

#### 2.3.2. Prior level 2

The standard deviations $\sigma_{\beta }=(\sigma_{\beta 1},\sigma_{\beta 2})$ , $\sigma_{\gamma }=(\sigma_{\gamma 1},\sigma_{\gamma 2})$ and $\sigma_{\theta }=(\sigma_{\theta 1},\sigma_{\theta 2})$ have independent exponential priors Exp(2) . The exponential prior was chosen because using an informative prior for group-level variance is recommended, particularly when the number of groups is small.^21^ Additionally, we examined other non-informative priors as part of a sensitivity analysis. Assuming similar effects across the subtrials, the prior was chosen to yield a 0.86 probability that each parameter is <1 , which is suitable when modelling the continuous outcome on the log scale. These parameters determine the extent of information sharing between subtrials. The lower boundaries for $\sigma_{\beta }$ , $\sigma_{\gamma }$ and $\sigma_{\theta }$ are constrained to 0.1 (ı.e. Exp(2) truncated at 0.1), to prevent convergence issues. Similarly to the stratified analysis, the prior means $\mu_{\beta }=(\mu_{\beta 1},\mu_{\beta 2})$ , $\mu_{\gamma }=(\mu_{\gamma 1},\mu_{\gamma 2})$ and $\mu_{\theta }=(\mu_{\theta 1},\mu_{\theta 2})$ have independent normal prior distributions $N(0,5^{2})$ . The standard deviation of the continuous outcome is assigned an inverse gamma prior $\sigma_{1}∼IG(0.5,0.005)$ . Here, we use a non-informative prior to minimise the sharing of information between the subtrials for this parameter. The correlation matrix is assigned an LKJ prior Ω∼LKJCorr(η) with a shape parameter η = 5.

### 2.4. Binary method for basket trials (BIN)

We compare the ABBA method with the BIN method, where we fit a logistic regression model to the dichotomised responder data, modelling the probability of response with the logistic regression

(2) $$\mathrm{logit}(p_{i})=\xi_{i}$$

where $\xi_{i}=\beta +\gamma \log(x_{i})+\theta t_{i}$ .

#### 2.4.1. Prior level 1

The parameters β , γ and θ have independent normal priors $N(0,5^{2})$ . For a basket trial, the BIN method models the binary responder data with a logistic regression that allows for the sharing of information between subtrials:

$$\xi_{i}=\beta_{k}+\gamma_{k}\log(x_{i})+\theta_{k}t_{i}$$

where $\beta_{k}$ , $\gamma_{k}$ and $\theta_{k}$ are the intercept, baseline effect and treatment effect for subtrial k . The priors for these parameters are:

$$\begin{array}{rl} \beta_{k} & ∼N(\mu_{\beta },\sigma_{\beta })\\ \gamma_{k} & ∼N(\mu_{\gamma },\sigma_{\gamma })\\ \theta_{k} & ∼N(\mu_{\theta },\sigma_{\theta }) \end{array}$$

#### 2.4.2. Prior level 2

The prior means $\mu_{\beta }$ , $\mu_{\gamma }$ and $\mu_{\theta }$ have independent normal priors $N(0,5^{2})$ . The standard deviations $\sigma_{\beta }$ , $\sigma_{\gamma }$ and $\sigma_{\theta }$ , which control the amount of information sharing between subtrials, have independent exponential priors Exp(2) truncated below at 0.3. Truncation (in both ABBA and BIN models) was initially applied due to the models’ failure to converge. For the ABBA model, truncating at 0.1 was sufficient to resolve the convergence issues. However, for the BIN model, truncation at both 0.1 and 0.2 still resulted in convergence problems, while truncating at 0.3 appeared to be effective.

### 2.5. Inferring success probability

For the ABBA method, the probability of success for participant i is the probability of being a responder inferred from the model:

$$\begin{array}{rl} r_{i} & =P(y_{i1}>\log(20)\wedge y_{i2}^{⋆}>0|\nu )=\int_{\log(20)}^{\infty }\int_{0}^{\infty }f_{Y_{1},Y_{2}^{⋆}}(y_{i1},y_{i2}^{⋆};\nu )dy_{i1}dy_{i2}^{⋆} \end{array}$$

where $f_{Y_{1},Y_{2}^{⋆}}(y_{1i},y_{2i^{⋆}};\nu )$ is the pdf of the bivariate normal distribution from equation (1), and ν is a vector of model parameters $\nu =(\beta ,\gamma ,\theta ,\mu_{\beta },\mu_{\gamma },\mu_{\theta },\sigma_{\beta },\sigma_{\gamma },\sigma_{\theta },\Omega ,\sigma_{1})$ .

For the BIN method, the probability of success for participant i is computed as follows:

$$r_{i}=P(y_{i}=1|\nu )=\exp\;(\xi_{i}|\nu )/(1+exp(\xi_{i}|\nu ))$$

where $\xi_{i}$ is the linear predictor from equation (2), and ν is a vector of model parameters $\nu =(\beta ,\gamma ,\theta ,\mu_{\beta },\mu_{\gamma },\mu_{\theta },\sigma_{\beta },\sigma_{\gamma },\sigma_{\theta })$ .

The log odds ratio for the k th subtrial, $\lambda_{k}$ , is computed as

$$\lambda_{k}=\sum_{i=1}^{n_{k}}\lambda_{i},$$

where $n_{k}$ is the number of participants in the k th subtrial. The individual log odds ratio for the i th participant is defined as

$$\lambda_{i}=\log\left(\frac{\left(r_{i,t})/(1-r_{i,t}\right)}{\left(r_{i,c})/(1-r_{i,c}\right)}\right)$$

where $r_{i,t}$ and $r_{i,c}$ denote the probabilities of success for participant i in the treatment and control arms, respectively.

### 2.6. Implementation

The Bayesian analysis models are implemented in Stan,^22^using the “rstan” package.^23^ For the multivariate probit regression, the latent continuous variable is divided into two parts based on whether the corresponding observed binary variable is 0 or 1. This division results in positive-constrained and negative-constrained components, where the sizes correspond to the counts of true (1) and false (0) observations in the binary variable. The positive-constrained and negative-constrained components are sampled separately from the uniform distribution, and the latent variable is constructed to account for the indices of 0s and 1s in the observed binary variable. To obtain the posterior distribution of the parameters, we employ two parallel chains, each running the Hamiltonian Monte Carlo sampler for 10,000 iterations after a burn-in period of 5,000 iterations. We examined the Markov Chain Monte Carlo (MCMC) output to ensure that no warnings indicated convergence issues or other sampling problems. R code for implementing the ABBA and BIN methods (with and without borrowing of information) is available on GitHub (https://github.com/svetlanache/ABBA).

## 3. Simulation study

To understand the properties of the ABBA model and compare it with the BIN model, we conducted a series of simulation studies. We investigated basket trials with three or six subtrials, assuming that participants were equally randomised. In most scenarios, we used a relatively small sample size (50 participants per subtrial) to reflect the typical sample sizes observed in real-world phase II clinical trials. To evaluate the method’s performance with larger samples, we also considered a sample size of 100 participants per arm for one of the scenarios, as well as a scenario with unequal sample sizes across the subtrials. Working with the posterior distribution of the log odds ratios λ = $\left(\lambda_{1},\ldots ,\lambda_{K}\right)$ , where K is the number of subtrials, we evaluate the following performance measures as recommended in Morris et al.^24^:

(i) Bias of the posterior means of λ , defined as the average distance from the true value of the log odds ratios, averaged across simulation replicates:
$$\mathrm{Bias}(\lambda )=\frac{1}{M}\sum_{m=1}^{M}{\bar{\lambda }}_{m}-\hat{\lambda }$$
  where M is the total number of simulation replicates, ${\bar{\lambda }}_{m}$ is the posterior mean of the log odds ratio for the m th replicate, and $\hat{\lambda }$ is the estimate of the true odds ratio, obtained using simulations with a sample size of 3,000,000.
(ii) Posterior precision of λ , defined as the reciprocal of the posterior variance of λ , averaged across simulation replicates:
$$\mathrm{Precision}(\lambda )=\frac{1}{M}\sum_{m=1}^{M}\frac{1}{\mathrm{Var}(\lambda_{m})}$$
  To evaluate the properties of the 95% high density interval (HDI) for λ , we consider:
(iii) one-sided power defined as the proportion of simulations where the lower limit of 95% HDI for λ is >0, averaged across simulation replicates:
$$\frac{1}{M}\sum_{m=1}^{M}1(\lambda_{m,low}>0)$$
  In null scenarios, this corresponds to the type I error rate, which is theoretically controlled at 2.5%.
(iv) Width of 95% HDI for λ , averaged across simulation replicates:
$$\frac{1}{M}\sum_{m=1}^{M}(\lambda_{m,up}-\lambda_{m,low})$$
(v) Coverage, defined as the proportion of simulation replicates in which the 95% HDI for λ includes the true value:
$$\frac{1}{M}\sum_{m=1}^{M}1(\lambda_{m,low}\leq \hat{\lambda }\leq \lambda_{m,up})$$
  Table 1 summarises the simulation scenarios, including the parameters used and characteristics of the simulated data.

**Table 1.** True values of the parameters for simulation studies, as estimated for a sample size of 3,000,000.

Scenario	Subtrial	LOR	RR ${}_{c}$	RR ${}_{t}$	$\beta_{1}$	$\beta_{2}$	$\gamma_{1}$	$\gamma_{2}$	$\theta_{1}$	$\theta_{2}$	$\sigma_{1}$	$\sigma_{2}$
1	1	0.00	0.20	0.20	1.07	0.04	0.5	–0.1	0.0	0.0	0.5	1
	2	0.00	0.20	0.20	1.07	0.04	0.5	–0.1	0.0	0.0	0.5	1
	3	0.00	0.20	0.20	1.07	0.04	0.5	–0.1	0.0	0.0	0.5	1
2	1	1.35	0.19	0.39	0.84	0.39	0.5	–0.1	0.7	0.0	0.5	1
	2	1.35	0.19	0.39	0.84	0.39	0.5	–0.1	0.7	0.0	0.5	1
	3	1.35	0.19	0.39	0.84	0.39	0.5	–0.1	0.7	0.0	0.5	1
3	1	0.67	0.24	0.38	2.91	0.22	0.0	–0.1	0.0	1.0	0.5	1
	2	0.67	0.24	0.38	2.91	0.22	0.0	–0.1	0.0	1.0	0.5	1
	3	0.67	0.24	0.38	2.91	0.22	0.0	–0.1	0.0	1.0	0.5	1
4	1	1.25	0.19	0.39	0.93	0.18	0.5	–0.1	0.5	0.3	0.5	1
	2	1.25	0.19	0.39	0.93	0.18	0.5	–0.1	0.5	0.3	0.5	1
	3	1.25	0.19	0.39	0.93	0.18	0.5	–0.1	0.5	0.3	0.5	1
5	1	1.21	0.20	0.38	0.24	0.52	0.7	–0.2	0.4	0.4	0.5	1
	2	1.21	0.20	0.38	0.24	0.52	0.7	–0.2	0.4	0.4	0.5	1
	3	0.00	0.27	0.27	0.56	0.56	0.7	–0.2	0.0	0.0	0.5	1
6	1	0.00	0.30	0.30	1.15	0.00	0.5	0.0	0.0	0.0	0.5	1
	2	0.00	0.30	0.30	1.15	0.00	0.5	0.0	0.0	0.0	0.5	1
	3	1.13	0.23	0.44	1.04	–0.17	0.5	0.0	0.4	0.4	0.5	1
7	1	0.71	0.28	0.37	0.90	0.94	0.5	–0.1	0.5	–0.5	0.5	1
	2	1.02	0.22	0.37	0.90	0.43	0.5	–0.1	0.5	0.0	0.5	1
	3	1.43	0.20	0.46	0.98	0.18	0.5	–0.1	0.5	0.5	0.5	1
8	1	-0.58	0.21	0.18	1.28	1.11	0.5	–0.4	–0.5	0.8	0.5	1
	2	0.70	0.19	0.33	1.14	1.16	0.5	–0.4	0.0	0.8	0.5	1
	3	1.70	0.19	0.50	1.14	1.18	0.5	–0.4	0.5	0.8	0.5	1

LOR: log odds ratio; RR ${}_{c}$ : response rate in the control arm; RR ${}_{t}$ : response rate in the treatment arm. The correlation parameter ρ was set to 0.3 in all scenarios.

We investigated two settings: one with a global effect, in which there is either the presence or absence of an effect, and another with non-global effects, in which there is a mixture of presence and absence of effects. The global effect setting comprised scenarios 1–4:

Scenario 1: The null scenario (no treatment effect in all subtrials).

Scenario 2: Treatment effect on the continuous component only, consistent across subtrials.

Scenario 3: Treatment effect on the latent component only, consistent across subtrials.

Scenario 4: Treatment effect on both components, consistent across subtrials.

The non-global effect setting was explored through scenarios 5–8:

Scenario 5: Treatment effect on both components consistent in two subtrials, no treatment effect in the other subtrial. Hereafter, subtrials with no treatment effect will be referred to as null subtrials.

Scenario 6: Treatment effect on both components in one subtrial, no treatment effect in the other two subtrials.

Scenario 7: Treatment effect consistent on the continuous component, inconsistent on the latent component.

Scenario 8: Treatment effect consistent on the latent component, inconsistent on the continuous component.

Additional simulations presented in the Supplemental material included scenarios with larger values for the standard deviation and scenarios with a greater number of subtrials. We also conducted a sensitivity analysis by considering a scenario in which the data-generating mechanism differed from the model. In this case, we used a multivariate skew-normal distribution.^25^ For each scenario, we simulated 1,000 replicates. (For the null scenario, we simulated 5,000 replicates. For a true type I error rate of 0.05, this would produce a Monte Carlo standard error for the estimated type I error rate of 0.003.) To simulate the data, the parameter vectors for the baseline effect γ and the treatment effect θ were fixed. The vectors of intercepts β were optimised to achieve a target response rate, which was 0.2 for the null scenarios, and 0.2 and 0.4 for the control and treatment arms, respectively, in the non-null scenarios (optimisation was performed using the optim function in R). In all scenarios, the correlation parameter ρ was set to 0.3, inducing a mild correlation between the outcome components. To compare the proposed methodology with the analysis that does not borrow information between subtrials, we also conducted a stratified analysis, examining each subtrial separately for scenarios presented in Table 1. As part of a sensitivity analysis, we analysed a representative scenario (scenario 4, which has a consistent treatment effect across the subtrials for both components) using different priors for the standard deviation $\sigma_{\beta }$ , $\sigma_{\gamma }$ and $\sigma_{\theta }$ (see Supplemental material).

## 4. Results

Table 2 summarises the width of the 95% HDI for the log odds ratio and the power for scenarios presented in Table 1, for the ABBA method with sharing of information (ABBAs, where the addition of the letter ‘s’ indicates sharing) compared to the BIN method with sharing of information (BINs). The ABBAs method demonstrates a reduction of 12% to 25% in the width of the 95% HDI for the log odds ratio, and an increase in power of up to 146%. The type I error rate, as assessed in Scenario 1 (the global null scenario), is well controlled at 2.5%. For Scenario 4, where the treatment effect is consistent across the subtrials, we simulated additional data with 100 participants per arm and achieved powers of 0.65, 0.66, and 0.64 for the BINs model – approximately 25% lower than the powers achieved with the ABBAs model using 50 participants per arm (0.9, 0.86, and 0.85). Thus, the increase in power with the ABBA model is equivalent to more than a 50% reduction in sample size. In heterogeneous scenarios (Scenarios 5 and 6), some inflation in the type I error rate is observed in the null subtrials for both ABBA and BIN methods, as expected when information borrowing occurs. Although the inflation is greater with the ABBAs method compared to the BINs method, the ABBAs method also yields a more substantial increase in power for the non-null subtrials in these scenarios. In Scenario 8, where the continuous component is inconsistent across subtrials, ABBA maintains control of the nominal type I error rate for the null subtrial, whereas the BIN method shows notable type I error inflation and reduced coverage.

**Table 2.** Mean 95% HDI for the log odds ratios and one-sided power for subtrials in a basket trial.

		95% HDI for LOR			Power		
Scenario	Subtrial	ABBAs	BINs	Δ	ABBAs	BINs	Δ
1	1	−0.92 to 0.9	−1.23 to 1.17	25%	0.01	0.01	⋆
	2	−0.91 to 0.9	−1.23 to 1.18	25%	0.01	0.01	⋆
	3	−0.92 to 0.89	−1.23 to 1.17	25%	0.01	0.01	⋆
2	1	0.53 to 2.31	0.05 to 2.26	20%	0.91	0.54	68%
	2	0.51 to 2.29	0.02 to 2.24	20%	0.88	0.54	65%
	3	0.51 to 2.29	0.01 to 2.23	19%	0.89	0.52	72%
3	1	−0.09 to 1.55	−0.31 to 1.78	21%	0.40	0.25	58%
	2	−0.1 to 1.54	−0.34 to 1.75	21%	0.40	0.24	68%
	3	−0.1 to 1.54	−0.32 to 1.76	21%	0.40	0.24	63%
4	1	0.45 to 2.19	0.06 to 2.28	22%	0.90	0.56	61%
	2	0.44 to 2.18	0.03 to 2.25	22%	0.86	0.56	55%
	3	0.44 to 2.18	0.03 to 2.24	21%	0.85	0.54	57%
5	1	0.22 to 2.02	−0.25 to 2.01	20%	0.71	0.29	140%
	2	0.2 to 2.01	−0.27 to 1.99	20%	0.70	0.30	139%
	3	−0.62 to 1.19	−0.83 to 1.46	21%	0.09	0.08	⋆
6	1	−0.7 to 1.01	−0.93 to 1.27	22%	0.04	0.03	⋆
	2	−0.71 to 1	−0.95 to 1.26	22%	0.03	0.03	⋆
	3	0.02 to 1.78	−0.36 to 1.85	20%	0.51	0.22	129%
7	1	0.06 to 1.85	−0.38 to 1.77	17%	0.56	0.22	147%
	2	0.16 to 1.93	−0.23 to 1.97	20%	0.67	0.35	94%
	3	0.35 to 2.17	0.05 to 2.24	18%	0.82	0.54	51%
8	1	−1.33 to 0.73	−0.92 to 1.6	19%	0.01	0.08	⋆
	2	−0.14 to 1.76	−0.27 to 2.01	17%	0.39	0.32	23%
	3	0.72 to 2.62	0.21 to 2.51	17%	0.95	0.65	46%

HDI: high-density interval; ABBA: augmented binary method for basket trials; BIN: binary method for basket trials; ABBAs: ABBA with sharing; BINs: BIN with sharing; LOR: log odds ratio. ${}^{⋆}$ In scenario 1, a type I error rate is controlled at 2.5% for both the ABBAs and the BINs methods. In scenario 5 (subtrial 3) and scenario 6 (subtrials 1), there is a slight inflation in the type I error rate for ABBAs compared to BINs, while in scenario 12 (subtrial 1), there is a reduction in the type I error rate for ABBAs compared to BINs.

To evaluate the properties of the ABBA and BIN models without borrowing information between subtrials (ABBAns vs. BINns, where “ns” indicates non-sharing), we conducted separate analyses for each subtrial (stratified analysis) for scenarios presented in Table 1. Table 3 presents the 95% HDI for the log odds ratio and the power. The ABBAns method results in a reduction of 21% to 31% in the width of the 95% HDI across all scenarios, and an increase in power of 33% to 192% in non-null scenarios. Scenario 1 (the global null) shows that though the type 1 error rate is controlled at 2.5% for the BINns method, there is some inflation of the type I error for the ABBAns (3.0%). In scenarios with heterogeneous subtrials (Scenarios 6 and 5), some inflation of the type 1 error rate occurs in the null subtrials; however, a substantial increase in power is observed in the non-null subtrials in these scenarios.

**Table 3.** Mean 95% HDI for the log odds ratios and one-sided power for the stratified analysis (ABBAns and BINns: no information sharing).

		95% HDI for LOR			Power		
Scenario	Subtrial	ABBAns	BINns	Δ	ABBAns	BINns	Δ
1	1	− 1.15 to 1.14	− 1.75 to 1.55	31%	0.03	0.02	⋆
	2	− 1.13 to 1.15	− 1.73 to 1.55	30%	0.03	0.02	⋆
	3	− 1.16 to 1.13	− 1.74 to 1.53	30%	0.03	0.02	⋆
2	1	0.36 to 2.61	− 0.23 to 2.68	23%	0.74	0.34	115%
	2	0.34 to 2.58	− 0.28 to 2.64	23%	0.73	0.35	110%
	3	0.32 to 2.57	− 0.31 to 2.58	22%	0.72	0.34	112%
3	1	− 0.21 to 1.78	− 0.56 to 2.15	27%	0.34	0.21	62%
	2	− 0.22 to 1.77	− 0.61 to 2.11	27%	0.33	0.18	77%
	3	− 0.22 to 1.77	− 0.58 to 2.14	27%	0.32	0.19	68%
4	1	0.29 to 2.49	− 0.22 to 2.7	25%	0.71	0.36	96%
	2	0.27 to 2.47	− 0.27 to 2.65	25%	0.70	0.36	97%
	3	0.26 to 2.46	− 0.29 to 2.59	24%	0.69	0.35	95%
5	1	0.24 to 2.47	− 0.34 to 2.59	24%	0.66	0.30	118%
	2	0.21 to 2.44	− 0.4 to 2.53	24%	0.66	0.29	126%
	3	− 1.1 to 1.07	− 1.48 to 1.31	22%	0.03	0.02	⋆
6	1	− 1.02 to 1.05	− 1.39 to 1.35	24%	0.04	0.03	⋆
	2	− 1.03 to 1.04	− 1.42 to 1.32	24%	0.03	0.03	⋆
	3	0.17 to 2.31	− 0.28 to 2.45	22%	0.62	0.36	71%
7	1	− 0.3 to 1.8	− 0.84 to 1.82	21%	0.28	0.10	192%
	2	0.03 to 2.18	− 0.55 to 2.3	24%	0.53	0.23	131%
	3	0.49 to 2.66	− 0.01 to 2.8	23%	0.81	0.51	57%
8	1	− 1.55 to 0.78	− 1.78 to 1.45	28%	0.01	0.02	⋆
	2	− 0.21 to 1.93	− 0.51 to 2.4	26%	0.35	0.25	40%
	3	0.71 to 2.92	0.32 to 3.13	21%	0.90	0.68	33%

HDI: high-density interval; ABBAns: augmented binary method for basket trials with non-sharing; BINns: binary method for basket trials with non-sharing; LOR: log odds ratio. ${}^{⋆}$ In scenario 1, scenario 5 (subtrial 3), scenario 6 (subtrials 1 and 2), scenario 8 (subtrial 1) and scenario 12 (subtrial 1), the power represents the type I error rate.

Table 4 compares the ABBA method with and without sharing of information (ABBAs vs. ABBAns), as well as the BIN method with and without sharing of information (BINs vs. BINns). Sharing information between subtrials results in a reduction of 12% to 21%/18% to 27% in the width of the 95% HDI for the log odds ratio. There is a notable decrease in the type I error rate for Scenario 1 and an increase in power for most scenarios. In subtrial 3 in Scenario 6 sharing of information shows a decrease in power for both methods, though the latent variable method achieves a smaller decrease. In Scenario 7, where the treatment effect for the latent component is inconsistent between the subtrials, information sharing in the latent variable model results in a slight reduction in power for subtrial 3.

**Table 4.** Reduction in 95% HDI for the log odds ratios and increase in power for the ABBAs method with information sharing versus the stratified ABBA method (ABBAns), and for the BINs method with information sharing versus the stratified BIN method (BINns).

		ABBAs versus ABBAns		BINs versus BINns	
		Reduction	Increase	Reduction	Increase
Scenario	Subtrial	in 95% HDI	in power	in 95% HDI	in power
1	1	21%	⋆	27%	⋆
	2	21%	⋆	27%	⋆
	3	21%	⋆	26%	⋆
2	1	21%	23%	24%	57%
	2	21%	21%	24%	54%
	3	21%	24%	23%	52%
3	1	17%	17%	23%	21%
	2	17%	23%	23%	30%
	3	17%	23%	23%	26%
4	1	21%	26%	24%	54%
	2	21%	23%	24%	55%
	3	21%	25%	23%	55%
5	1	19%	7%	23%	− 3%
	2	19%	7%	23%	1%
	3	16%	⋆⋆	18%	⋆⋆
6	1	17%	⋆⋆⋆	20%	⋆⋆⋆
	2	17%	⋆⋆⋆	20%	⋆⋆⋆
	3	18%	−16 %	19%	−38 %
7	1	15%	96%	19%	132%
	2	18%	27%	23%	51%
	3	17%	1%	22%	6%
8	1	12%	#	22%	#
	2	12%	10%	22%	26%
	3	14%	5%	18%	−4 %

HDI: high-density interval; ABBA: augmented binary method for basket trials; ABBAs: ABBA with sharing; ABBAns: ABBA with non-sharing; BIN: binary method for basket trials; BINs: BIN with sharing; BINns: BIN with non-sharing. ${}^{⋆}$ Scenario 1. A reduction in type I error rate for methods with information sharing: 66%, 65% and 57% for ABBAs; 58%, 52% and 47% for BINs. ${}^{⋆⋆}$ Scenario 5, subtrial 3. An inflation in type I error rate for methods with sharing: ABBAs = 0.09, ABBAns = 0.03; BINs = 0.08, BINns = 0.02. ${}^{⋆⋆⋆}$ Scenario 6, subtrials 1 and 2. No change in type I error rate for methods with and without information sharing: ABBAs and ABBAns = 0.04; BINs and BINns = 0.03. ${}^{\#}$ Scenario 8, subtrial 1. An inflation in type I error rate for BINs (0.08) compared to BINns (0.02). No change in type I error rate for the ABBA method with and without information sharing (0.01). ${}^{\#\#}$ Scenario 12, subtrial 1. An inflation in the type I error rate for ABBAs (0.09) compared to ABBAns (0.03). An inflation in type I error rate for BINs (0.2) compared to BINns (0.02).

**Figure 1.**
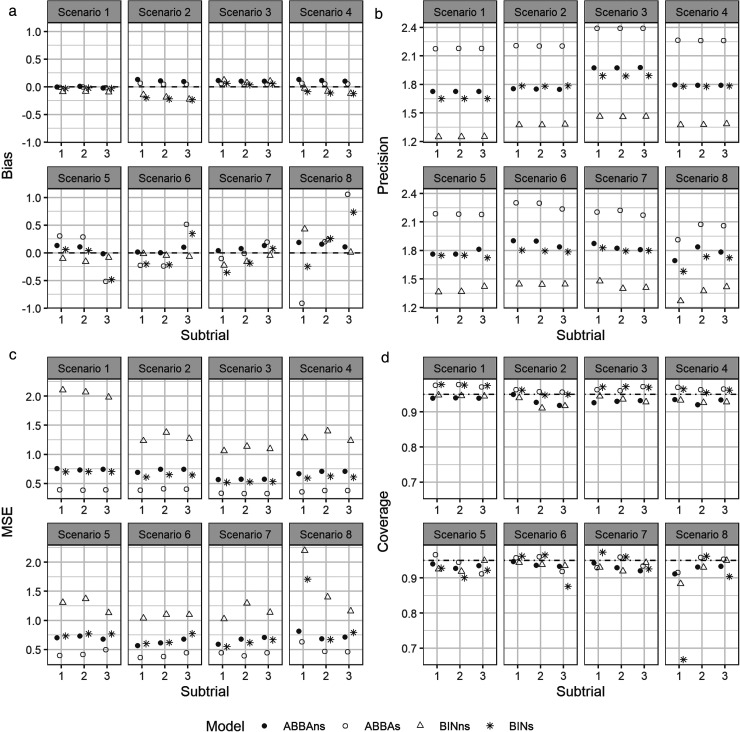
(a) Bias, (b) precision, (c) mean squared error, (d) coverage, of the posterior estimators for the log odds ratios. ABBAs/BINs – the methods with information sharing; ABBAns/BINns – the methods without information sharing (stratified analysis).

Figure 1 compares the bias (panel A), precision (panel B), mean squared error (panel C) and coverage (panel D) of the posterior mean for the log odds ratio, for the ABBA and BIN models with and without information sharing. It demonstrates that the ABBA model with information sharing (ABBAs) produces equivalent or smaller bias across nearly all scenarios (panel A), and substantially higher precision (panel B) and lower mean squared error (panel C) in all scenarios. The coverage for the ABBAs method (panel D) is approximately nominal for Scenarios 1–4, where the treatment effect is consistent across subtrials. However, for Scenarios 5–8, the coverage is below nominal for some subtrials. For Scenario 5, the lowest coverage (91.2%) occurs in subtrial 3, where there is no treatment effect, while the treatment effect is consistent across both components in the other two subtrials. A consistent treatment effect on the latent component only (Scenario 8) results in a slight drop in coverage for the ABBAs method, but a substantial drop for the BINs method in the null subtrial, where the components of the treatment effect act in opposite directions. A similar pattern was observed in the only non-null subtrial in Scenario 6.

## 5. Real data analysis

To illustrate the performance of the models on real data, we conducted a re-analysis of data from two clinical trials. This study, carried out under YODA Project 2023-5145, used data obtained from the Yale University Open Data Access Project, which has an agreement with Janssen Research & Development, L.L.C. The interpretation and reporting of research using this data are solely the responsibility of the authors and do not necessarily represent the official views of the Yale University Open Data Access Project or Janssen Research & Development, L.L.C. In both clinical trials, we examined outcomes related to DAS28 and ACR-N.

In NCT01645280 (EudraCT NUMBER: 2011-001122-18, Protocol CNTO1275ARA2001),^26^patients were randomised into five groups: placebo ( n=55 ), two groups receiving ustekinumab (different regiments, n=55 each), and guselkumab (different regiments, n=55 and 54). The primary endpoint was the proportion of patients achieving at least ACR20 at week 28, that is, the proportion of responders according to the ACR20 criteria. Patients were classified as non-responders if no ACR component data were available at week 28 or if they initiated prohibited medications (including glucocorticoids for rheumatoid arthritis), increased the methotrexate or glucocorticoid dose above the baseline level, or discontinued the study agent for any reason.

We compared the disease activity score-28 using CRP (DAS28-CRP) at 28 weeks between usetkinumab (combining two dose groups) and placebo arms in the primary analysis population. If patients had data for at least one DAS28-CRP component at week 28, missing components were imputed using the last observation carried forward method. In the latent variable model, DAS28-CRP at week 28 represented a continuous component of the outcome, while an indicator of drug withdrawal or administration of prohibited medications represented a binary component. In the binary model, patients were classified as responders if the drug was not withdrawn during the study, no prohibited medications were administered, and DAS28-CRP at week 28 was below the threshold of 2.6. We adjusted for DAS28-CRP at baseline in the analyses. For the ACR-N outcome, we computed the ACR-N score at 28 weeks and adjusted the analysis for the baseline value of the DAS28-CRP measure. In the binary model for ACR-N, patients were classified as responders if their ACR-N score exceeded 20%.

In NCT01077362 (EudraCT NUMBER: 2009-012265-60, Protocol CNTO1275PSA3002), 312 adults with active psoriatic arthritis were randomised to different doses of ustekinumab or placebo with crossover to ustekinumab.^27^ We compared DAS28-CRP at week 24 between the ustekinumab and placebo arms in the primary analysis population. The outcomes were specified similarly to those for the NCT01645280 trial (see Table 5).

**Table 5.** Number of samples in the exemplar clinical trials, tabulated by the binary indicator and threshold for the DAS28-CRP measure.

		DAS28-CRP		ACR-N	
Trial	Group	<2.6	≥ 2.6	>0.2	≤ 0.2
NCT01645280 ( N=151 )	Control ( N=50 )	**5**	42	**25**	22
		0	3	1	2
	Treatment ( N=101 )	**16**	79	**50**	45
		0	6	1	5
NCT01077362 ( N=268 )	Control ( N=81 )	**28**	45	**29**	44
		1	7	1	7
	Treatment ( N=187 )	**70**	96	**87**	79
		3	18	6	15

DAS28-CRP: disease activity score-28 with C-reactive protein; ACR-N: American College of Rheumatology-N. The numbers in bold represent the number of responders for the analysis with the binary model.

The resulting 95% HDI for the log odds ratios are presented in Table 6 for a hypothetical basket trial that combines the two trials (combined analysis), and for each trial analysed separately (stratified analysis). For NCT01645280 and NCT01077362, sharing of information in the ABBA model resulted in a 5% and 7% decrease in the width on the 95% HDI for the DAS28 outcome, and 4% and 3% for the ACR-N outcome, respectively. The ABBAs model offers a substantial advantage in reducing the width of the 95% HDI (19% to 39%), compared to the BINs model.

**Table 6.** 95% HDI for the log odds ratios for the real data analysis.

		Analysis	95% HDI		
Outcome	Trial	type	ABBA	BIN	Δ
DAS28	NCT01645280	Stratified	−0.3 to 1.07	−0.54 to 1.7	39%
		Combined	−0.13 to 1.17	−0.47 to 1.37	29%
	NCT01077362	Stratified	0.01 to 0.93	−0.026 to 0.94	23%
		Combined	−0.03 to 0.83	−0.22 to 0.93	25%
ACR-N	NCT01645280	Stratified	−0.43 to 0.65	−0.74 to 0.62	21%
		Combined	−0.36 to 0.68	−0.64 to 0.64	19%
	NCT01077362	Stratified	0.19 to 1.07	0.14 to 1.24	20%
		Combined	0.15 to 0.99	0.07 to 1.15	22%

HDI: high-density interval; ABBA: augmented binary method for basket trials; BIN: binary method for basket trials; DAS28: disease activity score-28; ACR-N: American College of Rheumatology-N. The Δ 95% HDI represents the percentage decrease in the width of the 95% HDI for the ABBA model relative to the BIN model. For NCT01645280 and NCT01077362, sharing of information in the ABBA model resulted in a 5% and 7% decrease in the width of the 95% HDI for the DAS28 outcome, and a 4% and 3% decrease for the ACR-N outcome, respectively.

## 6. Discussion

In this paper, we propose and assess an extension of the augmented binary method to basket trials (ABBA). The method shares information for composite outcomes by leveraging the augmented binary approach via a latent variable framework. The underlying assumption is that a continuous latent variable determines the observed binary component, which we jointly model with the observed continuous component. This method draws motivation from the augmented binary method, which previously demonstrated superior performance in non-basket rheumatology trials compared to standard analyses of binary outcomes. We conducted various comparisons of our ABBA method against standard methods and demonstrated its substantial advantages: it achieves a notable reduction in the width of the 95% HDI for the log odds ratio, along with increased power and precision compared to standard logistic regression methods that do not utilise the continuous nature of outcomes.

When comparing the ABBA method with information borrowing between subtrials to the method without borrowing, we observed a substantial reduction in the width of the 95% HDI across various simulation scenarios. Our simulation study further highlighted that the ABBA method exhibited the highest precision for the posterior mean of the log odds ratio compared to other methods, even when the data-generating mechanism differed from that of the model (Supplemental material). In some scenarios where the treatment effect varied inconsistently across subtrials, the ABBA method performed comparably to the BIN method in controlling the type I error rate and achieving coverage close to the nominal level. While careful consideration is necessary regarding the expected consistency of effects across subtrials, ABBA proved advantageous when the treatment effect was consistent for the latent component but inconsistent for the continuous component. We note that our method requires simulations to determine the sample size rather than analytical calculations. Our simulations considered relatively small sample sizes because they reflect the realistic sample sizes of phase II trials. The sample size chosen resulted in a well-controlled type I error. Additionally, there was an increase in power observed in comparison with the standard binary power calculation, which was 0.47, as computed with the *pwr.2p2n.test* function in the *pwr* R package for a single subtrial ( n=25 per arm) and response rates of 0.2 and 0.4 for the control and treatment arms, respectively. Further work could involve analytical calculation of the required sample sizes, guided by the Bayesian sample size determination approach.^28^

We illustrated the application of our method to real data from rheumatology trials. Although the trials were conducted separately, we hypothesised a scenario resembling a basket trial comprising two subtrials due to the investigation of the same treatment and common clinical outcomes. We investigated how combining the two trials into a basket trial might have affected the results. The ABBA model achieved a substantial reduction in the width of the 95% HDI compared to the analysis of binary outcomes with sharing information between subtrials. However, the reduction achieved by ABBAs was minor compared to that by ABBAns. This can be attributed to our hypothesised basket trial comprising only two subtrials, thereby limiting the amount of expected information sharing.

**Figure 2.**
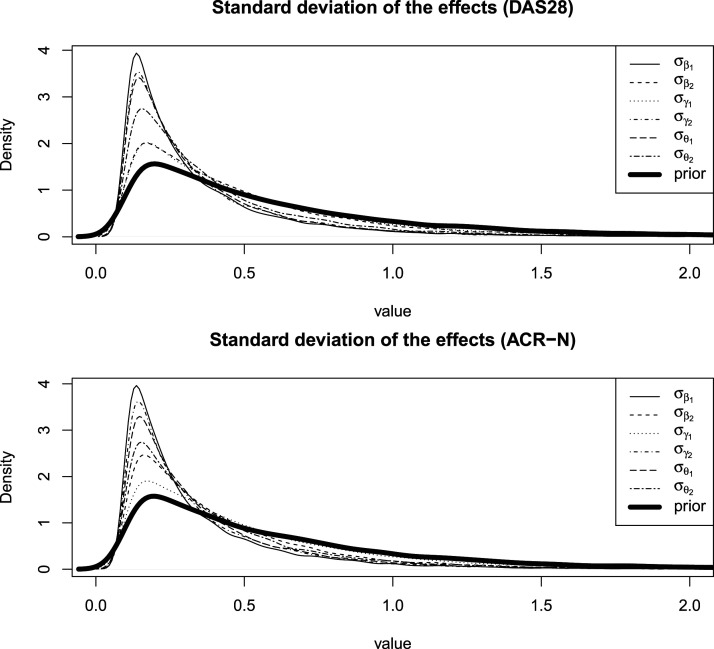
Posterior and prior distributions of the standard deviation of the effects.

Our method has a few limitations, such as longer computational time and occasional convergence issues of the MCMC algorithm. Another issue is a prior specification, especially for the variation between subtrials. With a small number of subtrials, the data contribute minimally to the posterior distribution, which is primarily influenced by the prior (Figure 2). Thus, the choice of the prior for this parameter can significantly impact the degree of information borrowing across subtrials.^14,21,29^

In this study, we utilised an exponential prior that assigns a higher probability to smaller values of the parameter. Future research will explore alternative priors such as commensurate prior, which relies on distributional discrepancy to measure commensurability between subtrials and borrows information from those with the most similar treatment effect.^17^ Our model assumes exchangeability, which, while reasonable in some clinical areas, might be too restrictive. Further work to revise the model by relaxing the exchangeability assumption, thus allowing more flexible sharing of information, would be valuable. Investigating the ABBA method for different models that account for heterogeneity between the subtrials, such as the EXNEX model,^30^could lead to a more efficient analysis of the data. Our method assumes that the clinical outcome is the same in each disease. This is not always the case, as different conditions in a basket trial often have different clinical outcomes. However, in this case, there might be a common secondary outcome (e.g. a mechanistic biomarker) that is the same for each condition. This setting is particularly relevant, but not limited to, clinical trials in rheumatology. We therefore aim to investigate basket trials involving distinct outcomes where information may be shared via a common secondary outcome. In conclusion, the ABBA makes more efficient use of data by borrowing information across subtrials, presenting a promising avenue for further research.

## Supplemental Material

sj-pdf-1-smm-10.1177_09622802251403365 - Supplemental material for Augmented binary method for basket trials (ABBA) Supplemental material, sj-pdf-1-smm-10.1177_09622802251403365 for Augmented binary method for basket trials (ABBA) by Svetlana Cherlin and James M S Wason in Statistical Methods in Medical Research
