# Supplementary material for: Augmented binary method for basket trials (ABBA)
Source: Stat Methods Med Res. 2025 Dec 5;35(1):172–85. doi: 10.1177/09622802251403365 (PMC12824627; doi:10.1177/09622802251403365)
Supplement: sj-pdf-1-smm-10.1177_09622802251403365 - Supplemental material for Augmented binary method for basket trials (ABBA) [file sj-pdf-1-smm-10.1177_09622802251403365.pdf]

Supplementary material for “Augmented Binary Method for Basket Trials (ABBA)”

Journal Title
XX(X):1–4
©The Author(s) 2021
Reprints and permission:
sagepub.co.uk/journalsPermissions.nav
DOI: 10.1177/ToBeAssigned
www.sagepub.com/
SAGE

Svetlana Cherlin and James M S Wason

Additional simulation scenarios

We conducted simulation scenarios (Table 1) that investigate the behaviour of the model for data simulated with larger values for the standard deviation, as well as for a greater number of subtrials.

Scenario S1: Scenario with three subtrials of equal size (n = 50 per subtrial). Treatment effect on both components, consistent across subtrials.The data were generated using larger values for the standard deviations of the continuous and latent components.

Scenario S2: Scenario with six subtrials. Treatment effect on both components, consistent across subtrials. We consider two possibilities: subtrials of equal size (n = 50 per subtrial; Scenario S2a), and subtrials of unequal size (n = {50, 46, 42, 38, 34, 30}; Scenario S2b).

Scenario S3: Scenario with six subtrials of unequal size (n = {50, 46, 42, 38, 34, 30}). Treatment effect on both components, consistent across subtrials.

Scenario S4: Scenario with six subtrials of equal size (n = 50 per subtrial). Treatment effect on both components is inconsistent across subtrials.

The ABBA method demonstrates a reduction of 12% - 25% in the width of the 95% HDI for the log odds ratio, and an increase in power of up to 80% (Table 2). In Scenario S4, where the continuous component is inconsistent across subtrials, ABBA maintains control of the nominal type I error rate for the null subtrial, whereas the BIN method shows notable type I error inflation and reduced coverage. Sharing information between subtrials results in a reduction in the width of the 95% HDI for the log odds ratio, as well an increase in power, compared with stratified analysis (Tables 3 and 4).

Table 1. True values of the parameters for simulation studies, as estimated for a sample size of 3,000,000. LOR: log odds ratio, RR_c: response rate in the control arm, RR_t: response rate in the treatment arm. The correlation parameter ρ was set to 0.3 in all scenarios.

Scenario	Subtrial	LOR	RR_c	RR_t	β_1	β_2	γ_1	γ_2	θ_1	θ_2	σ_1	σ_2
S1	1	0.62	0.24	0.35	0.93	0.18	0.5	-0.1	0.5	0.3	1.0	2
	2	0.62	0.24	0.35	0.93	0.18	0.5	-0.1	0.5	0.3	1.0	2
	3	0.62	0.24	0.35	0.93	0.18	0.5	-0.1	0.5	0.3	1.0	2
S2	1	1.25	0.19	0.39	0.93	0.18	0.5	-0.1	0.5	0.3	0.5	1
	2	1.25	0.19	0.39	0.93	0.18	0.5	-0.1	0.5	0.3	0.5	1
	3	1.25	0.19	0.39	0.93	0.18	0.5	-0.1	0.5	0.3	0.5	1
	4	1.25	0.19	0.39	0.93	0.18	0.5	-0.1	0.5	0.3	0.5	1
	5	1.25	0.19	0.39	0.93	0.18	0.5	-0.1	0.5	0.3	0.5	1
	6	1.25	0.19	0.39	0.93	0.18	0.5	-0.1	0.5	0.3	0.5	1
S3	1	0.00	0.15	0.15	0.93	0.18	0.5	-0.1	0.0	0.0	0.5	1
	2	2.59	0.20	0.72	0.93	0.18	0.5	-0.1	1.0	1.0	0.5	1
	3	1.62	0.16	0.38	0.93	0.18	0.5	-0.1	0.5	0.5	0.5	1
	4	2.57	0.20	0.73	0.93	0.18	0.5	-0.1	1.0	1.0	0.5	1
	5	1.64	0.18	0.41	0.93	0.18	0.5	-0.1	0.5	0.5	0.5	1
	6	1.41	0.17	0.39	0.93	0.18	0.5	-0.1	1.0	0.0	0.5	1

Table 2. Mean 95% HDI for the log odds ratios and one-sided power for subtrials in a basket trial. * In Scenario S3 (subtrial 1), there is a reduction in the type I error rate for ABBA compared to BIN.

Scenario	Subtrial	95% HDI for LOR			Power		
		ABBA	BIN	Δ	ABBA	BIN	Δ
S1	1	-0.14 to 1.44	-0.41 to 1.71	25%	0.33	0.19	75%
	2	-0.16 to 1.43	-0.43 to 1.69	25%	0.34	0.19	80%
	3	-0.16 to 1.43	-0.43 to 1.69	25%	0.34	0.18	83%
S2a	1	0.55 - 2.03	0.17 - 2.1	24%	0.96	0.69	40%
	2	0.57 - 2.04	0.18 - 2.12	24%	0.96	0.68	40%
	3	0.56 - 2.03	0.18 - 2.11	24%	0.96	0.70	37%
	4	0.56 - 2.03	0.19 - 2.12	24%	0.96	0.70	35%
	5	0.55 - 2.03	0.18 - 2.11	24%	0.96	0.70	38%
	6	0.55 - 2.02	0.18 - 2.12	24%	0.96	0.70	37%
S2b	1	0.52 - 2.05	0.15 - 2.14	23%	0.95	0.66	44%
	2	0.51 - 2.08	0.14 - 2.18	23%	0.93	0.65	43%
	3	0.49 - 2.09	0.09 - 2.19	23%	0.91	0.61	50%
	4	0.45 - 2.1	0.06 - 2.21	23%	0.92	0.57	61%
	5	0.44 - 2.15	0.03 - 2.24	23%	0.90	0.56	59%
	6	0.42 - 2.19	0.03 - 2.3	22%	0.88	0.57	55%
S3	1	-0.63 to 1.32	-0.62 to 1.94	24%	0.09	0.20	⋆
	2	1.57 - 3.54	1.13 - 3.43	14%	1.00	1.00	0%
	3	0.67 - 2.48	0.38 - 2.54	16%	0.96	0.80	20%
	4	1.56 - 3.54	1.12 - 3.41	14%	1.00	1.00	0%
	5	0.66 - 2.47	0.37 - 2.54	16%	0.95	0.78	21%
	6	0.52 - 2.47	0.21 - 2.43	12%	0.86	0.67	27%

Table 3. Mean 95% HDI for the log odds ratios and one-sided power for the stratified analysis. *In Scenario S3 (subtrial 1), the power represents the type I error rate.

Scenario	Subtrial	95% HDI for LOR			Power		
		ABBA	BIN	Δ	ABBA	BIN	Δ
S1	1	-0.33 to 1.7	-0.68 to 2.05	25%	0.25	0.15	64%
	2	-0.35 to 1.69	-0.73 to 2.01	26%	0.27	0.15	78%
	3	-0.37 to 1.68	-0.72 to 2.01	25%	0.26	0.15	74%
S2a	1	0.26 - 2.46	-0.27 to 2.64	25%	0.69	0.35	94%
	2	0.29 - 2.5	-0.25 to 2.66	24%	0.73	0.36	103%
	3	0.29 - 2.49	-0.25 to 2.64	24%	0.70	0.36	94%
	4	0.29 - 2.49	-0.24 to 2.66	24%	0.70	0.38	84%
	5	0.27 - 2.47	-0.26 to 2.65	24%	0.67	0.36	86%
	6	0.28 - 2.48	-0.24 to 2.7	25%	0.70	0.38	84%
S2b	1	0.26 - 2.46	-0.27 to 2.62	24%	0.69	0.35	98%
	2	0.24 - 2.55	-0.3 to 2.75	24%	0.66	0.34	96%
	3	0.18 - 2.61	-0.41 to 2.81	24%	0.62	0.28	126%
	4	0.09 - 2.66	-0.52 to 2.92	25%	0.54	0.26	113%
	5	0.04 - 2.82	-0.65 to 3.09	26%	0.53	0.24	125%
	6	-0.01 to 2.99	-0.68 to 3.42	27%	0.49	0.22	120%
S3	1	-1.12 to 1.12	-1.78 to 1.62	34%	0.03	0.02	⋆
	2	1.75 - 4.25	1.26 - 4.38	20%	1.00	0.98	2%
	3	0.54 - 2.73	0.03 - 2.91	24%	0.84	0.51	64%
	4	1.75 - 4.26	1.25 - 4.38	20%	1.00	0.98	2%
	5	0.52 - 2.72	0.02 - 2.92	24%	0.83	0.51	61%
	6	0.25 - 2.61	-0.32 to 2.62	20%	0.66	0.32	106%

Table 4. Reduction in 95% HDI for the log odds ratios and increase in power for the ABBA method with information sharing vs the stratified ABBA method (ABBAs), and for the BIN method with information sharing vs the stratified BIN method (BINs). *Scenario S3, subtrial 1. An inflation in type I error rate for ABBA (0.09) compared to ABBAs (0.03). An inflation in type I error rate for BIN (0.2) compared to BINs (0.02).

Scenario	Subtrial	ABBA vs ABBAs		BIN vs BINs	
		Reduction in 95% HDI	Increase in power	Reduction in 95% HDI	Increase in power
S1	1	22%	33%	23%	25%
	2	22%	28%	23%	26%
	3	22%	31%	22%	25%
S2a	1	33%	40%	34%	94%
	2	33%	32%	34%	90%
	3	33%	37%	33%	94%
	4	33%	36%	34%	85%
	5	33%	43%	34%	93%
	6	33%	37%	34%	84%
S2b	1	31%	36%	31%	88%
	2	32%	40%	33%	91%
	3	34%	46%	35%	121%
	4	36%	68%	38%	123%
	5	38%	69%	41%	139%
	6	41%	78%	45%	152%
S3	1	13%	*	25%	*
	2	21%	0%	26%	2%
	3	17%	14%	25%	55%
	4	21%	0%	26%	2%
	5	17%	15%	25%	53%
	6	17%	29%	24%	109%

Sensitivity analysis

Different data generating mechanism

We conducted a sensitivity analysis by considering a scenario in which the data-generating mechanism differs from the model. In this scenario, we used a multivariate skew-normal distribution with the parameter regulating the amount of skewness set to $\{1, 1\}$. This scenario consists of three subtrials of equal size (n = 50 per subtrial), with the treatment effect on both components being consistent across subtrials. The analysis was based on 1,000 replicates.

Table 5 presents the parameters used in the simulations. Table 6 presents the results for the ABBA and BIN models, with the ABBA model outperforming its comparator. Table 7 demonstrates improved operating characteristics achieved through information sharing. Figure 1 shows the bias, precision, mean squared error and coverage for different models. Although the coverage for the ABBA method is below nominal, the mean squared error is the lowest, while the power and precision are the highest among the competing models.

Table 5. Parameters for simulation studies. LOR: log odds ratio, RR_c: response rate in the control arm, RR_t: response rate in the treatment arm, as estimated for a sample size of 3,000,000.

Subtrial	LOR	RR_c	RR_t	β_1	β_2	γ_1	γ_2	θ_1	θ_2	σ_1	σ_2	ρ
1	0.64	0.09	0.15	0.93	0.18	0.5	-0.1	0.5	0.3	0.5	1	0.3
2	0.64	0.09	0.15	0.93	0.18	0.5	-0.1	0.5	0.3	0.5	1	0.3
3	0.64	0.09	0.15	0.93	0.18	0.5	-0.1	0.5	0.3	0.5	1	0.3

Table 6. Mean 95% HDI for the log odds ratios and one-sided power for subtrials with and without information sharing.

Analysis type	Subtrial	95% HDI for LOR			Power		
		ABBA	BIN	Δ	ABBA	BIN	Δ
Information sharing	1	0.19 - 2.03	-0.1 to 2.06	15%	0.65	0.44	49%
	2	0.21 - 2.06	-0.1 to 2.07	15%	0.70	0.43	63%
	3	0.19 - 2.03	-0.1 to 2.07	15%	0.65	0.43	51%
Stratified analysis	1	0.09 - 2.23	-0.39 to 2.41	24%	0.55	0.28	95%
	2	0.13 - 2.28	-0.38 to 2.42	23%	0.59	0.30	100%
	3	0.1 - 2.24	-0.39 to 2.44	24%	0.55	0.28	100%

Table 7. Reduction in 95% HDI for the log odds ratios and increase in power for the ABBA method with information sharing vs the stratified ABBA method (ABBAs), and for the BIN method with information sharing vs the stratified BIN method (BINs).

Subtrial	ABBA vs ABBAs		BIN vs BINs	
	Reduction in 95% HDI	Increase in power	Reduction in 95% HDI	Increase in power
1	14%	18%	23%	54%
2	14%	19%	23%	45%
3	14%	17%	23%	55%

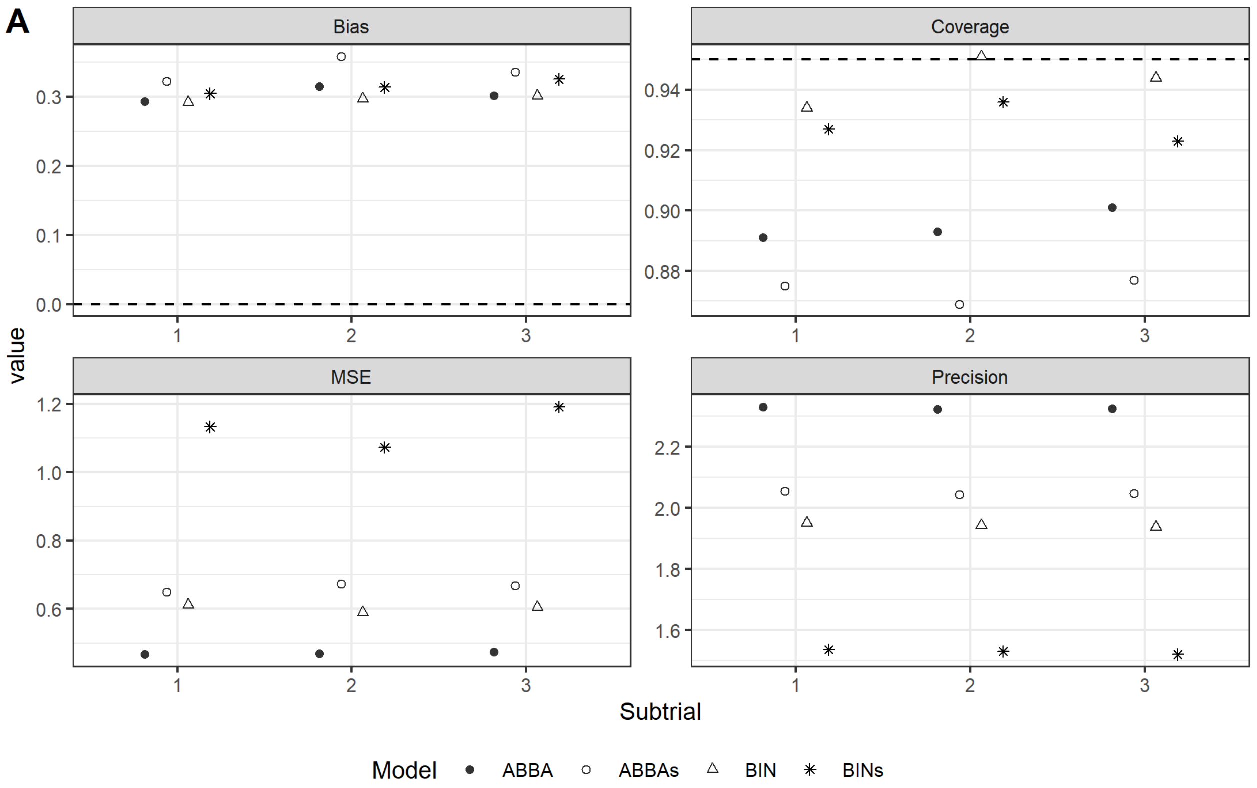

Figure 1. Operating characteristics based on posterior estimates of log-odds ratios for the sensitivity analysis.

Prior

We analysed a representative scenario (scenario 4 in Table 1 of the main text, which has a consistent treatment effect across the subtrials for both components) using different priors for the standard deviation σ_β, σ_γ and σ_θ: half-normal(0,1) (truncated at 0.1 for ABBA and at 0.3 for BIN) and half-t (df = 1, location = 0.1, scale = 0.5). The results were similar for the half-normal and half-t priors, with a comparable decrease in the width of the 95% HDI and an increase in power relative to the exponential prior (Table 8).

Table 8. Mean 95% HDI for the log odds ratios and one-sided power for Scenario 4 analysed with exponential, half-normal (HN) and half-t priors. The parameters for the half-t prior are: Half-t(l=0.1,s=0.5,df=1), where l, s and df are the location, scale and degrees of freedom, respectively.

Prior	Subtrial	95% HDI for LOR		Δ	Power		Δ
		ABBA	BIN		ABBA	BIN	
Exp(2)	1	0.45 - 2.19	0.06 - 2.28	22%	0.90	0.56	61%
	2	0.44 - 2.18	0.03 - 2.25	22%	0.86	0.56	55%
	3	0.44 - 2.18	0.03 - 2.24	21%	0.85	0.54	57%
HN(0,1)	1	0.42 - 2.23	0.02 - 2.34	22%	0.86	0.51	69%
	2	0.4 - 2.22	-0.01 to 2.31	22%	0.83	0.52	60%
	3	0.4 - 2.21	-0.02 to 2.29	21%	0.82	0.51	60%
Half-t	1	0.44 - 2.2	0.04 - 2.31	22%	0.89	0.55	62%
	2	0.43 - 2.19	0.01 - 2.28	22%	0.85	0.54	56%
	3	0.43 - 2.19	0.01 - 2.27	22%	0.85	0.53	61%
